# Modification of an inpatient medical management protocol for pediatric Avoidant/Restrictive Food Intake Disorder: improving the standard of care

**DOI:** 10.1186/s40337-023-00895-9

**Published:** 2023-09-22

**Authors:** Sasha Gorrell, Siena S. Vendlinski, Arianna S. Thompson, Amanda E. Downey, Rachel Kramer, Lisa Hail, Sharon Clifton, Sarah Forsberg, Erin E. Reilly, Elizabeth Saunders, Sara M. Buckelew, Daniel Le Grange

**Affiliations:** 1 Department of Psychiatry and Behavioral Sciences, University of California, San Francisco, 675 18th St., San Francisco, CA 94143 USA; 2 Department of Pediatrics, University of California, San Francisco, San Francisco, CA USA; 3 School of Medicine, University of California, San Francisco, San Francisco, CA USA; 4 Department of Psychiatry and Behavioral Neuroscience, The University of Chicago, Chicago, IL USA

**Keywords:** ARFID, Eating disorders, Inpatient protocol, Inpatient treatment, Adolescents

## Abstract

**Background:**

No guidelines currently exist that represent a standardization of care for Avoidant/Restrictive Food Intake Disorder (ARFID) on an inpatient service. Unique features of this diagnosis (e.g., sensory sensitivity contributing to involuntary emesis) suggest that established protocols that were developed for anorexia nervosa might be less effective for adolescents with ARFID. To inform improved inpatient medical stabilization and care for these patients, we first provide an overview of clinical characteristics for patients with ARFID who presented to a pediatric hospital for inpatient eating disorder care. We use these descriptives to outline the rationale for, and executions of, modifications to an inpatient protocol designed to flexibly meet the needs of this clinical population.

**Methods:**

Chart review with descriptive statistics were conducted for patients who had received an ARFID diagnosis from March 2019 to March 2023 (*N* = 32, aged 9–23). We then present a case series (*n* = 3) of adolescents who either transitioned to a novel adjusted protocol from an original standard of care on the inpatient service, or who received only the standard protocol.

**Results:**

The sample was aged *M*(*SD*) = 15.6 (3.3) years, 53% male, and a majority (69%) presented with the ARFID presentation specific to fear of negative consequences. On average, patients had deviated from their growth curve for just over two years and presented with mean 76% of their estimated body weight. Of those requiring nasogastric tube insertion during admission (*n* = 8, 25%), average duration of tube placement was 15 days. From within this sample, case series data suggest that the adjusted protocol will continue to have a positive impact on care trajectory among adolescents admitted for ARFID including improved weight gain, reduction of emesis, and improved food intake.

**Conclusions:**

Findings demonstrate the likely need to tailor established medical inpatient protocols for those with ARFID given different symptom presentation and maintenance factors compared to patients with anorexia nervosa. Further research is warranted to explore the longer-term impact of protocol changes and to inform standardization of care for this high priority clinical population across care sites.

## Abstract (plain language summary)

No current standard of care exists for pediatric patients with Avoidant/Restrictive Food Intake Disorder (ARFID) who are hospitalized for medical stabilization related to complications secondary to malnutrition. Clinical features of this diagnosis (e.g., sensory sensitivity) suggest that existing treatment protocols developed for patients with other restrictive eating disorders, like anorexia nervosa, may be less effective for patients with ARFID. This study first describes a pediatric sample of patients with ARFID upon admission to an inpatient service. Then, a case series is used to illustrate the potential benefits of using an adjusted protocol that was modified to better suit the needs of children and adolescents with ARFID. Findings support future study of the proposed adjusted protocol and may inform future standardization of improved care for this high priority clinical population.

## Introduction

Avoidant/Restrictive Food Intake Disorder (ARFID) is characterized by restriction and avoidance of food intake due to sensory sensitivity, lack of hedonic drive to approach food, or fear of negative consequences unrelated to body image concerns [1]. These eating disturbances can result in nutritional deficiency, weight loss or failure to achieve appropriate weight gain, and significant interference with psychosocial function. Formally recognized in the diagnostic nosology for the past decade, gathering evidence suggests clinical presentations of ARFID are heterogeneous and often inclusive of significant psychiatric comorbidity [2, 20]. Moreover, while more research is required to accurately capture prevalence rates for this disorder on a population level, a recent review estimates that rates can range from 0.3 to 15.5% in community samples, with even higher estimates in specialized treatment settings for eating disorders (5–55.5%) [20].

In addition to these reported incidence rates in pediatric tertiary care, evidence suggests that a significant subset of individuals with ARFID require medical hospitalization during the course of treatment (based on criteria from Society for Adolescent Health and Medicine [SAHM] guidelines; [3, 14]). The clinical presentation of ARFID may bring about unique challenges to tolerating existing refeeding protocols—developed for the treatment of other eating disorders, such as anorexia nervosa [7]—while hospitalized. For example, sensory sensitivity and intolerance of food volume may lead to pronounced gastrointestinal distress and involuntary emesis [13], which might compromise nutrition and facilitation of weight gain. Indeed, some work has shown that when compared to patients hospitalized who are diagnosed with anorexia nervosa, patients diagnosed with ARFID demonstrate slower weekly weight gain [13], rely more on enteral nutrition, and require longer hospitalizations [21]. Despite these preliminary indications, to date, a more comprehensive understanding of the clinical presentation of inpatients who are diagnosed with ARFID has been limited by the heterogeneous nature of this disorder, within relatively limited sample sizes.

Improving inpatient outcomes for patients with ARFID depends on more effective characterization of this sample and use of these data to drive innovations in protocols for optimal support. However, in a recent survey of physicians, only 22.7% reported having a specialized protocol for ARFID [8]. Although no current guidelines exist to date that represent a standardization of care for ARFID on an inpatient service, a recent case series of patients (*N* = 16, *M*_age_ = 11.5 years, 75% female) suggests that due in part to the heterogeneity in symptoms of this patient population, a multidisciplinary, medical, and behavioral treatment model was effective for a variety of clinical presentations [11].

To inform the future development of a protocol that might be standardized and implemented across care sites, we present an adjusted care model for pediatric ARFID that has recently been implemented at a large urban children’s hospital with a dedicated inpatient eating disorder service (described just below and in Table 2). First, in Study 1, we present a characterization of patients with ARFID to contextualize the medical and psychiatric presentation of this population beyond that of several existing studies [11, 13, 21]. These data both illustrate the severity of this clinical population, as well as provide a foundational rationale for adjusting our current standard of care to better meet the clinical needs of this population. In Study 2, we then describe three cases where patients either transitioned to an adjusted protocol from original standard of care during the same hospital admission or received only the standard protocol, with particular challenge.

### Study 1: Characterization of inpatients with ARFID

#### Participants

Participants for both Study 1 and Study 2 include patients who had been hospitalized on an inpatient medical stabilization unit for medical complications secondary to malnutrition and received a diagnosis of ARFID from March 2019-March 2023 (*N* = 32, aged 9–23). Chart review was conducted for all data acquisition. This work was granted approval and participant consent was waived by the University of California, San Francisco (UCSF) Institutional Review Board (# 23-38777).

#### Chart review

Data were extracted from the electronic medical system by two independent coders (authors SSV and AST). Years of inclusion were determined based on the significant increase in average daily census (more than double) just prior to the COVID-19 pandemic, resulting in initial attempts to adapt care to support a more heterogenous population. Only patients with a confirmed diagnosis of ARFID (determined at the time of admission based on a clinical assessment conducted by psychologists specializing in eating disorders) were included. Patients who had an ARFID diagnosis at intake but later were diagnosed with anorexia nervosa were excluded from the current study. The three ARFID presentations described were operationalized according to the descriptions originally included in the Diagnostic and Statistical Manual of Mental Disorders [1] and typically assessed with standardized measurements (e.g., Nine Item Avoidant/Restrictive Food Intake Screen, [2]). Based on these characterizations, determination of ARFID presentations were drawn from assessment notes entered by the clinical psychology provider. Any uncertainty regarding presentation classification from chart review was confirmed by provider recall (authors AD, SF, RK). If the patient had more than one hospitalization on our service for malnutrition or eating disorder-related cause, data were extracted for the most recent admission. Lab results and vital signs were recorded from values drawn within the first day (24 h) of admission. Estimated body weight (EBW) was calculated by registered dietitians, based on historic growth records; values for this study were recorded from the initial dietitian note typically entered on the first day of admission. Further, information about current psychological treatment, comorbid diagnoses, and suicidal ideation (as measured by the National Institutes of Health Ask Suicide-Screening Questions Tool ASQ) were drawn from chart records.

#### Statistical analyses

Within the analytic sample used to characterize our patient population with ARFID in this timeframe (Study 1; *N* = 32), we conducted descriptive statistics to calculate frequencies, means, and standard deviations for admission weight and weight history, vital signs, and serum laboratory values, as these are clinical indicators of the degree of medical instability. SPSS v29 was used for all analyses.

#### Results

Patients (*N* = 32) ranged in age from 9–23, *M*(*SD*) = 15.6 (3.3) years. The sample was nearly equally represented across sex (*n* = 17, 53% male), with all but one who identified as cisgender male or female (*n* = 1 transgender male). Race and ethnicity were reported as White (*n* = 12, 37.5%), Asian (*n* = 4, 12.5%), Black/African American (*n* = 3, 9.4%), or other (*n* = 13, 40.6%); just under half (*n* = 14, 43.8%) reported identifying as Latinx/Hispanic. Length of stay ranged from 2 to 47, *M*(*SD*) = 12.5 (9.7) days. Prior hospitalization for consequences of malnutrition was reported by *n* = 11 (34.4%) patients. Twelve patients presented with only one presentation of ARFID (37.5%) and *n* = 4 (12.5%) presented with all three; all others reported two presentations. Further descriptive statistics for clinical variables of interest can be found in Table 1.

**Table 1:** Descriptive statistics (*N* = 32)

Variable	Range	*n* (%)	Mean (SD)
*ARFID diagnostic subtype*			
Sensory sensitivity		15 (46.9)	
Fear of adverse consequences		22 (68.8)	
Low interest/appetite		18 (56.3)	
Estimated length of illness (months)	0–60.0		6.9 (12.9)
Time since growth curve deviation (months)	1.1–104.7		29.5 (28.0)
*Weight status at admission*			
BMI	8.4–20.3		15.5 (2.2)
%Estimated body weight	59.0–94.0		76.0 (7.4)
Nasogastric tube placed during admission		8 (25)	
Days with nasogastric tube during admission	1–44		14.75 (14.2)
Psychotropic medication prior to admission		10 (31.3)	
Escitalopram		4 (12.5)	
Mirtazapine		1 (3.1)	
Other medication		6 (18.8)	
*Ask Suicide-Screening Questions (ASQ)*			
Negative		22 (68.8)	
Non-acute positive		6 (18.8)	
Positive		0 (0)	
Missing		4 (12.5)	
*Comorbid psychiatric diagnosis*			
Generalized anxiety disorder		27 (84.4)	
Major depressive disorder		15 (46.9)	
Post-traumatic stress disorder		2 (6.3)	
Autism spectrum disorder		6 (18.8)	
Attention-deficit hyperactivity disorder		5 (15.6)	
Substance use disorder		2 (6.3)	
*Pre-admission treatment*			
Cognitive-behavioral therapy		0 (0)	
Family-based treatment		1 (3.1)	
Higher level of care		3 (9.4)	
Occupational therapy		2 (6.3)	
Other		7 (21.9)	
*Heart rate*			
Supine heart rate	38.0–107.0		75.45 (15.5)
Standing heart rate	72.0–162.0		112.2 (22.2)
Orthostatic heart rate change	5.0–88.0		36.7 (16.8)
Heart rate nadir	38.0–96.0		61.5 (14.0)
*Blood pressure*			
Supine systolic	76.0–125.0		101.6 (9.7)
Standing systolic	73.0–125.0		102.1 (11.9)
Systolic nadir (within first 24 h)	67.0–109.0		91.2 (10.3)
Supine diastolic	49.0–86.0		61.0 (8.7)
Standing diastolic	42.0–86.0		65.1 (10.7)
Diastolic nadir (within first 24 h)	39.0–72.0		54.2 (8.5)
*Serum markers*			
Sodium	130.0–143.0		138.2 (2.6)
Potassium	2.8–10.0		4.2 (1.2)
Magnesium	1.2–2.9		2.1 (0.27)
Phosphorus	2.8–5.9		3.7 (0.63)
Glucose	53.0–154.0		97.0 (24.7)
Zinc	45.0–135.0		68.1 (19.6)
Vitamin D	9.0–62.0		24.8 (12.7)

Unless otherwise noted, all variables represent values at admission

### Study 2: case studies

#### Description of the inpatient program

The inpatient service described here is located within a tertiary care hospital serving publicly and privately insured young people across the state of California. The adolescent medicine service is embedded on a general medical/surgical floor and interdisciplinary care is delivered by providers from the departments of nursing, psychiatry, pediatrics, nutrition, and social work. All patients and families receive interdisciplinary assessments and treatment plans while admitted, and disposition recommendations upon discharge. Family-based treatment (FBT) [12] is the prevailing treatment model once discharged, informing the philosophy and structure of programming while admitted. That is, caregivers are provided with psychoeducation about FBT (e.g., encouraged to take an agnostic view of the disorder) and are empowered to support their child during the admission to the extent that they are able to. Specifically, when appropriate, parents may supervise meals and snacks, and are encouraged to support and coach their children to complete the food provided. Admission eligibility is determined based on Society of Adolescent Health and Medicine (SAHM) criteria [14], and average length of stay for patients with transdiagnostic eating disorders is 10 days. A standard refeeding protocol is used for all patients on the service, typically those with anorexia nervosa (described more fully in Table 2). In the majority of cases, patients are started on a 2000 kilocalorie diet with supervised meals and snacks selected by a diet technician with little direct input from the youth or their caregivers [7]. Patients are offered meals and snacks that, if not completed, are replaced calorie for calorie with a liquid nutritional supplement; nasogastric tubes are used if patients are unable to complete food and nutrition supplement. Calorie amounts are typically advanced at a rate of 200 kilocalories/day, with adjustments made based on progress towards medical stability.

**Table 2:** Standard protocol for inpatient refeeding and adjusted protocol for patients with Avoidant/Restrictive Food Intake Disorder

	Standard protocol	Adjusted protocol
Overlap in protocol	Unique aspect of standard protocol	Unique aspect of adjusted protocol
*Caloric goals and monitoring*		
1. At admission, initiate 2,000 kcal diet unless determined to be high risk for refeeding syndrome	1. Protocol of increasing calories daily, calorie counting, and having meals determined by diet technician remains constant	1. Once medically stable enough, patients are transitioned from standard protocol to modified
2. Increase by 200 kcal every day until at estimated goal kcal		
3. Modify rate and amount of calorie increase as needed to support medical stabilization		
*Meal structure*		
1. 3 meals and 3 snacks provided daily	1. No use of commode/bathroom within 15 min after meals	1. Meal observations (and commode privileges) may be more flexible (e.g., patients may not need observation after meals or have to wait to use bathroom)
2. 30 min meals, 20 min snacks, 15 min nutritional supplement (if food is not completed)		
3. Patient care attendant observes meal/snack and observes after meal/snack for 15 min		
4. Family members encouraged to provide mealtime support. If caregiver is available and deemed safe, asked to fill in meal supervision role during later part of admission		
*Meal content*		
1. Meals and snacks are delivered by hospital kitchen	1. Meal content determined by diet technician based on available foods and calorie goal for the day	1. Meal content determined by caregiver and patient, if appropriate. Family provided with regular hospital menu, caloric goal and menu planning worksheet. Menus repeated daily as appropriate with modifications made to support increasing calorie goals
	2. No dietary modifications made except in case of food allergies, religious needs, and longstanding vegetarianism predating onset of illness. Vegan diets not accommodated	2. Dietary modifications allowed to preference intake of solid nutrition as long as fitting within close range of calorie goals
*Calorie/meal replacement*		
1. Percentage of meal eaten recorded by patient care attendant. Any nutrition not consumed via food is supplemented with nutritional beverage with a 1–1 cal replacement		1. Nursing staff can modify amount of replacement if patient ate mostly high caloric density or low caloric density foods
2. If all nutrition (food and supplement) not completed, supplement is given through nasogastric tube		2. If patient has aversion to supplement, alternate meal replacement items can be used if available to decrease reliance on nasogastric tube
3. Nasogastric tube feeds are completed directly after unfinished meal or snack		
4. Nasogastric tube is removed after 24 h of completing all nutrition by mouth		

#### Indication for protocol modification

As ARFID became more widely recognized and represented in the medical inpatient setting, several challenges with the standardized protocol emerged. Team members noted unique barriers to intake, driven by known characteristics of ARFID that were incompatible with the treatment approach. For example, many patients struggled with lack of access to preferred foods, had aversions to typically used nutrition supplements, including specific flavors, or had heightened sensitivity to physical sensations that triggered fear of vomiting and choking. In several cases, patients were unable to increase percentage of oral intake, relying predominantly on nasogastric tube feeding over the course of hospitalization, which led providers to weigh the risks and benefits of leaving the feeding tube in place upon discharge.

#### Protocol adjustments

A side-by-side comparison of the original and adjusted protocol is illustrated in Table 2. The adjusted protocol was developed over the prior two years with the aim of improving treatment outcomes in the hospital setting. The main differences between the two protocols are centered in three domains: (1) allowing patients and caregivers options to choose preferred foods, (2) greater flexibility with meal replacement, and (3) changes in supervision requirements. Case descriptions from preliminary use of the protocol described below illustrate successes and challenges with these changes; objective measures of treatment outcome, including patient, caregiver, and staff perspectives on feasibility and acceptability are forthcoming in a future publication.

The protocol was designed specifically for the picky/selective eating ARFID presentation; clinical observations suggested that patients with this particular presentation seemed to struggle most with pre-determined menus given their longstanding avoidance of several foods. However, as illustrated in the case examples, the adjusted protocol has since been applied more broadly to individuals with mixed ARFID presentations including fear of aversive consequences of eating and lack of interest in food/eating. Patients who were likely to accept a limited number of items from the protocolized menu that had been originally developed for patients with anorexia nervosa were considered suitable for the adjusted protocol. In addition, to trial an adjusted protocol, patients were required to be outside of the acute refeeding window and thus at low-risk for refeeding syndrome, not on electrolyte supplements due to biochemical refeeding syndrome, and considered able to safely tolerate some degree of variability in caloric intake. Given typical close coordination with caregivers in menu planning, parents needed to be willing/able to engage in menu planning with assistance, and thus were eligible if the patient could access menus in one of the available languages (English, Spanish, or Chinese) and interpretation of the menu in the family’s spoken language was feasible.

Once deemed appropriate, a team member reviewed the modifications with families. This included sharing the hospital menu that included calorie counts for food items, and information about the patient’s estimated caloric needs to support reversal of vital sign abnormalities and weight restoration. To facilitate collaborative meal planning, caregivers were asked to select all of the patient’s preferred foods on the menu, with patient collaboration when appropriate. From this list, the nurse coordinator created a menu that was repeated for several days. Meal trays were delivered 45 min before the scheduled mealtime. After the allotted meal time, a total calorie count for missed nutrition was determined by supervising nursing staff and a nutritional supplement was measured and provided to patients to replace (one-to-one) calories not consumed during the meal.

Although nutritional supplements were noted to be challenging and/or aversive for some patients, they were ultimately retained for use given ease of access for nursing staff. Alternate replacements to supplements (e.g., ice cream, graham crackers, items that were readily available on the floor) were allowed if considered to support increased intake or the patient had a specific aversion to the supplement drink. As per typical standard of care for all patients on the service, mealtimes were monitored with post-meal observations first by staff and later by parents, as appropriate. Behavior monitoring around bathroom use was flexible, and not enforced when determined to be safe per team consensus. Patient progress was operationalized by the percentage of meals and snacks completed, and the types of food items that were consumed was monitored throughout each day. The protocol efficacy was evaluated and if no progress was noted following two full days on the new protocol (e.g., minimal progress with oral intake) the team would consider resumption of the standard protocol.

Meal plan modification was also supported by team psychologists who met with patients and their family members 2–3 times per week with the aim of addressing other common barriers to eating. For example, patients and caretakers engaged in goal setting around food exposures (e.g., trying a bite of a new food, completing a target percentage of a meal), and in some cases families were encouraged to create a behavior plan with caregiver- and patient-identified rewards for progress (e.g., earning screen-time, a small prize, points towards an activity post-discharge). Common interventions included relaxation training, guided visualization, diaphragmatic breathing, mindfulness, cognitive reappraisal, and psychoeducation about ARFID and the mind–body connection to address common challenges including sensory sensitivity, misattribution of sensory sensations, pain, nausea and other somatic experiences, and anxiety-driven cognitions. Family members and patients were instructed on these skills alike.

### Patient 1: Patient on standard protocol for two consecutive admissions

Anya is a 10-year-old, Latina cisgender female residing with her mother, father and two older siblings. At the time of her first hospital admission, Anya presented with acute food refusal for one week, in the context of a reduction in appetite following exposure to a traumatic event. She had no known prior mental health history, and was described by her family as a “sensitive” child who had trouble with changes in routine and trying new things. Parents also described patterns of behavior consistent with separation anxiety that had intensified in the last week. She was admitted to the hospital with concerns for acute food refusal, a recent 10-pound weight loss and dehydration, and subsequently was found to have orthostatic tachycardia, hypotension, hypokalemia, and hypomagnesemia requiring repletion.

#### History of feeding/eating difficulties

Assessment by the mental health team indicated that Anya’s food and eating history was remarkable for longstanding sensory sensitivities (dislike of “chewy” foods that she found more difficult to swallow). Parents described difficulty encouraging Anya to eat foods the family enjoyed, and she endorsed a limited diet of pasta, rice, apple sauce, quesadillas with cheese, waffles, French fries and apple juice. She reported an aversion to most fruits, vegetables and meats, although she would eat corn, berries and bananas. Despite these food restrictions, Anya had no known history of medical complications or nutritional deficiencies; however, the family did report one prior episode of acute food refusal a few months prior to the admission that had resulted in an evaluation in the emergency room. At the time, Anya was unable to verbalize precipitants or fears associated with food refusal, later describing fear of vomiting. She had no prior mental health treatment history, and was diagnosed with generalized anxiety disorder during her hospital admission.

#### Course of treatment

Anya had two separate admissions two weeks apart, lasting 18 days and then 5 days respectively. She had historically tracked along the 25th BMI percentile for age and height until her first hospital admission, when she presented at the 10th BMI percentile and at 84% of her calculated EBW. She was started on the standard protocol but initially received 1400 kilocalories per day (instead of a typical 2000) given electrolyte disturbance suggestive of risk for refeeding syndrome. As a result, she initially lost weight over the first few days of this admission. She was noted to have difficulty taking any solid foods, resorting to primarily drinking fluids (milk or juice) and was unable to drink nutritional supplements due to concerns about upsetting her stomach. This led to the insertion of the nasogastric tube which was experienced as highly distressing to the patient, leading to a panic attack, gagging and vomiting, and requiring support from several staff and family members. Due to subsequent refusal of all food and beverages for two days, occupational therapy providers were consulted who then were able to support the family in devising a reward system to reinforce when Anya was able to try small bites of food. With consistent coaching from parents, and mental health and occupational therapy providers, Anya was able to gradually increase food intake, however the overall percentage of meals consumed remained minimal (10–15%). Anya complained of worsening abdominal pain with nasogastric feeds, an uncomfortable sensation in her throat that contributed to further reluctance to take nutrition by mouth, and had several episodes of emesis. She reported a general fear that “something bad is happening to my body”.

Despite weight gain (discharged from this first admission at 93% EBW) and resolution of medical stability, Anya did not progress with regards to solid food intake. Mother shared that Anya preferred the food that she makes at home (e.g., beef broth, rice, beans) and reported optimism that she would eat more with her mother's cooking. The team decided to leave the nasogastric tube in place at discharge with hope that she could gradually reduce her reliance.

Per typical post-discharge procedures, Anya subsequently presented for follow-up in the outpatient Adolescent Medicine clinic and was found to have pulled out her nasogastric tube two days prior, with improved solid food intake. However, laboratory findings were notable for hypoglycemia, dehydration and weight loss of 4 kg prompting immediate hospital readmission. The nasogastric tube was replaced and Anya continued to struggle to take nutrition by mouth, becoming agitated with symptoms of a panic attack at initiating of the tube feeds.

Once Anya’s vitals were stable for discharge, she again returned home with the nasogastric tube still in place; her parents managed these tube feeding times in a way that allowed Anya to return to school. They reported that she continued to improve oral food intake but noted that at times she would not finish her meal and ask parents to “connect her to the tube" instead. Due to improved intake, the nasogastric tube was ultimately removed 5 months after discharge. At her final visit approximately two years after initial hospital admission, the family reported Anya was eating everything provided and denied abdominal pain, bloating, choking, gagging, sensory difficulties. Anya was no longer engaged in psychotherapy, and her parents agreed that she seemed happy and engaged in school and developmentally normative activities. Her father noted some mild anxiety that sometimes impeded her ability to take in nutrition, but she was reportedly able to resolve her avoidance quickly. The family agreed they did not feel they needed additional medical or behavioral health support at that time.

### Patient 2: Patient who began with standard protocol and transitioned to adjusted protocol on day 18 during one admission

Azin is a 17-year-old, White, cisgender female residing with her mother and father, and two brothers; her older sister lives away from home at college. The family immigrated to the United States in 2017; Azin is bilingual, and her caregivers are monolingual Dari speakers. The family is Muslim and follow a halal diet. Prior to admissions, she had not participated in previous inpatient care or outpatient therapy and had no comorbid psychiatric conditions or medication use. Azin was referred by her pediatrician for further evaluation due to disordered eating concerns; she was first evaluated by our outpatient medical team in April 2021 with a BMI percentile = 0.27 (BMIz = − 2.78). The family worked to improve nutrition but Azin was ultimately admitted in July 2021 for low weight (BMI percentile =  < 1%, BMIZ = − 4.20) and orthostatic tachycardia; hospitalization lasted for 5 days. Azin was discharged with a plan of care that included recommendations for FBT that were unfulfilled. Azin again presented to outpatient clinic services in March 2023 with BMI Percentile, < 1%, BMIZ = − 5.29 and was subsequently admitted for the following 27 days.

#### History of feeding/eating difficulties

Azin met developmental milestones without significant delays. She did not have a history of significant medical concerns throughout childhood although both Azin and her mother noted that she had always been a picky eater with minimal interest in food who also has experienced early fullness and bloating after eating. Azin historically tracked at a low weight and per her mother’s report, has had consistent difficulty with weight gain.

#### Course of treatment

Upon her first admission, Azin was placed on standard protocol (i.e., starting at 2000 kilocalories, and increasing by 200 kilocalories per day) [7]. On the fourth day, Azin had one instance of involuntary emesis after breakfast which occurred again the day after and as her calories increased, she started to have more frequent emesis. On day 6, Azin was feeling so full she was not able to finish her meal or nutrition replacement and thus missed achieving total calorie needs; ultimately there were two other occasions where the team was unable to support the completion of nutritional supplements due to early fullness.

Azin shared that she rarely felt nauseated before meals on the unit but during meals would feel full and have difficulty eating once she felt this way. When presented with a nutritional supplement, she drank it willingly, yet she shared that she did not like the taste and drinking it exacerbated her fullness. For nearly all meals where she required a significant amount of supplement, Azin experienced emesis. Gastrointestinal specialists were consulted, and a gastric emptying study was performed with unremarkable results. Azin shared a consistent desire to gain weight in order to return home and that she noticed hunger, but struggled significantly with fullness. She also explained that she did not like the types of food the hospital offered; the family asked if food could be brought from home but, to date, this is not permissible within our service. There were several reasons why the team decided to implement the adjusted protocol on day 18. First, Azin was motivated to eat more and restore weight, yet the food we provided varied significantly from her family’s typical cuisine and foods she liked. Azin also experienced emesis after drinking the nutritional supplement 33% of days prior to protocol change (i.e., at least once per day on roughly a third of the days admitted). Average weight gain on this original protocol was 0.2 lbs per day (median and mode = 0).

#### Response to adjusted protocol

With her mother, Azin selected from the hospital menu provided to her by the head registered nurse and chose a balanced set of foods from all food groups. Azin completed 62% of her meals with food (completed the rest in supplements) after the first menu change. On day 21, Azin’s menus were updated again to add greater variety when she shared boredom in response to meal repetition; with this shift, food intake increased to 87%. She only had one episode of emesis in the 9 days following a change to the adjusted protocol (i.e., 11% of days), a significant improvement. When accounting for one day of significant weight loss, average weight gain was improved at 0.37 lbs per day (median = 0.55 lbs, mode = 0.88 lbs).

### Patient 3: Patient who began with standard protocol and transitioned to adjusted protocol at day 10 during one admission

Francisco is a 12-year-old Mexican–American cisgender male who resides in a small rural town with his biological parents (monolingual Spanish speaking) and several older siblings. As a toddler, he was diagnosed with developmental disability with associated developmental delays. In elementary school he was diagnosed with anxiety (including severe needle phobia) and autism spectrum disorder. Francisco is enrolled in special education with an Individualized Educational Plan, and his mother provides in home support services.

#### History of feeding/eating difficulties

Even as an infant, Francisco would not cry for food and his mother always had to anticipate when he might be hungry. Throughout his life, Francisco has been a selective and picky eater preferring to eat spaghetti, pizza, quesadillas, chicken, potatoes, chorizo, soup, oranges and bananas. Approximately 18-months prior to hospitalization, Francisco had a gastrointestinal illness with nausea and vomiting. While receiving medical care, he overheard parents being asked if he may have eaten any food that was “spoiled”. Following this appointment, he expressed fear that his food was “rotten” and subsequently restricted his already limited intake. Over time his restriction and avoidance behaviors escalated. Francisco increased the frequency of handwashing and began to avoid attending school fearing becoming sick away from home.

From age 4–8, Francisco’s weight for age percentile slowly decreased from the 75th to the 25th percentile, and linear growth falling below the curve likely associated with selective eating. Following the episode of illness, Francisco’s weight decreased more rapidly with a loss of 15% and he was admitted to the hospital at 62% of his EBW (calculated to be the 60th percentile). He was admitted to the hospital for low body weight, risk of refeeding syndrome, dehydration, and orthostasis.

#### Course of treatment

For the first 10-days of a 13-day admission, Francisco was on the standard meal protocol increasing from 1600 kilocalories (lower than typical starting point because his intake prior to admission was so minimal) to his goal intake of 3600 kilocalories per day. His parents reported surprise that he was able to eat foods that he had been avoiding, and he was willing to try new foods during the admission (e.g., grilled cheese); however, Francisco endorsed frustration that he was being “forced to try so many adult foods.” He enjoyed chocolate supplementation drinks though struggled with the volume of replacement required due to fullness. During the first half of the admission, he was also experiencing significant anxiety and distress related to his needle phobia requiring additional supports for blood draws.

#### Response to adjusted protocol

On day 10 after advancing to goal caloric intake of 3600 kilocalories, the nursing coordinator and psychologist met with Francisco’s mother to introduce the adjusted ARFID protocol with the support of a Spanish language interpreter. Mother was subsequently able to create appropriate meal and snack combinations to meet Francisco’s nutritional needs with minimal support. She was keenly aware of Francisco’s preferences and asked questions to see if those could be accommodated. For example, she knew that she wanted to add butter to each of his pancakes at breakfast, but that it would need to be melted to make the pancake less dry. Because the hospital has a limited number of his preferred foods, Francisco’s mother opted to order similar meals for each day with multiples of the same item (e.g., 2-grilled cheese sandwiches). Ideally, she would have liked to increase the amount of cheese and butter on a single sandwich, but that modification was not available within the ordering system.

A few days into hospital admission (while still on the standard meal protocol) Francisco’s mother shared her concern that the hospital was “too strict” and causing “trauma” for Francisco. She made a request to discharge as she was feeling reassured that he was eating more than he had been prior to admission. Mother did not consent to nasogastric tube placement but was amenable to offering alternate replacement options (e.g., ice cream) which Francisco refused because he was feeling too full; she was ultimately able to appreciate the risk of refeeding syndrome and agreed to continued admission.

Although he was relying heavily on nutritional supplements to meet his caloric needs, Francisco’s fear of vomiting related to eating was significantly reduced and he was no longer washing his hands excessively. On the adjusted protocol, his mother immediately demonstrated that she could plan meals and snacks to meet his energy needs with a clear grasp of how to increase density of his preferred foods. The family reported increased satisfaction and reduced distress with the opportunity to select preferred foods. Due to availability of transportation home and distress associated with being in the hospital, Francisco was discharged at a lower percent EBW than is standard, but family was successful in maintaining the rate of weight gain (3 pounds per week) for the two weeks following discharge. His mother identified practice menu planning in the hospital as helpful to clarify his daily caloric needs.

## Discussion

The current work is some of the first to explore the impact of implementing a modified refeeding protocol among young people hospitalized for medical complications secondary to malnutrition due to ARFID. In Study 1, our descriptive data highlight the severity of illness evidenced within this clinical population, including across heterogeneous presentations. Further, our case descriptions in Study 2 support the rationale for providing adjustments to our current clinical standard of care moving forward, for reasons that include (i) increasing the amount of nutrition and decreasing reliance on nasogastric tubes or nutritional supplements; and (ii) increasing patient and caregiver satisfaction, including reduction in distress.

Our descriptive data yielded some notable findings. Compared to a large systematic review of young people with ARFID in various care settings, our sample was slightly older and had a higher proportion of male-identifying patients [20]. The age discrepancy may be explained by the care provided by our tertiary care eating disorder program which cares for patients until the 26^th^ birthday. Almost half of our sample identified as Hispanic/Latinx, a sharp departure from other samples of inpatient youth with ARFID in which patients were predominantly White or ethnicity was not reported [13, 21, 22], reflecting the demographic makeup of our urban catchment region in the United States. Mental health comorbidities, namely anxiety disorders, were common in our sample, consistent with other epidemiological studies of young people with ARFID [3, 4, 10, 20, 22]. Autism spectrum disorder was also commonly observed in our sample as is found across diverse eating disorder treatment settings caring for youth with ARFID [20]. Like other samples of hospitalized youth with ARFID, the predominant presentation was fear of aversive consequences, though over half of participants demonstrated clinical characteristics common to more than one presentation. The symptom presentation of this population is difficult to generalize broadly owing to their heterogeneous clinical characteristics and etiologies, as well as nonspecific symptoms for which they may present to various medical specialists. Comprehensive education of all medical subspecialists and primary care providers is thus warranted to best detect these patients early in the course of treatment to prevent the sometimes irreversible consequences of prolonged malnutrition on growth and development [3].

In comparison with typical patient samples with anorexia nervosa, our ARFID sample was consistent with prior work in demonstrating a high representation of male patients [3, 5, 6, 17]. We note here that transdiagnostic eating disorders in males are more broadly under-recognized and under-treated, despite the growing number of affected males, which may account for the sex differences in prevalence [16, 15]. Compared to other inpatient youth with anorexia nervosa, mean illness duration was longer in our sample at 29.5 months, which may reflect the heterogeneous and sometimes nonspecific presenting symptoms of ARFID [5, 18]. Consistent with other studies, we found a higher burden of anxiety disorders and a lower burden of depressive disorders in the ARFID sample as compared to inpatient youth with anorexia nervosa [5, 17, 21]. Comparing the frequency and duration of nasogastric tube insertion in our sample with other samples of youth with anorexia nervosa and ARFID is difficult given heterogeneous practices regarding tube utilization. Experts in the field have urged caution in nasogastric tube placement in youth with ARFID given the risk of causing iatrogenic food aversions and other psychological consequences [9]; as such, decreased frequency and duration of nasogastric tube insertion is a priority for the modified ARFID protocol. Taken together, the unique characteristics and comorbidities of youth with ARFID argue for tailored, diagnosis-specific inpatient treatment protocols to best meet the needs of these patients and their caregivers.

Improving medical and psychological outcomes for medically hospitalized youth with ARFID requires thoughtful modification of the inpatient protocols used to treat hospitalized youth with other restrictive eating disorders, like anorexia nervosa. The case studies presented here highlight the heterogeneous psychiatric and medical symptoms which complicate the implementation of a “one-size-fits-all” protocolized approach. Case study 1 highlights the acute worsening of psychiatric comorbidities (anxiety and panic) resulting from a standardized nutrition plan without incorporating modifications that would reduce barriers to improvement. Specifically, prolonged nasogastric tube insertion, multiple hospitalizations, and other iatrogenic harms may have been avoided with the implementation of a patient-centered protocol allowing flexible nutritional choices. Case study 2 describes the somatic sensations that are often barriers to nutritional rehabilitation for youth with ARFID, a stark contrast to the strong body image disturbance typically driving food avoidance in those with anorexia nervosa. Working within a more flexible protocol offers youth agency in their nutritional rehabilitation plan as they navigate unique physical symptoms, providing the opportunity for a more synergistic partnership with the medical team towards the shared goal of weight restoration and associated medical stabilization. Case study 3 demonstrates how developmental delay and sensory challenges can impede success with a standard nutritional rehabilitation protocol, causing increased distress for both patients and caregivers. This patient and family ultimately left the hospital before discharge criteria were met and shortly after initiation of the modified protocol. Although it is therefore difficult to determine the impact of the adjusted protocol on this patient’s clinical outcomes when in hospital, the increased flexibility and caregiver autonomy over nutritional rehabilitation provided the caregiver the confidence to continue the recovery process safely at home. All three cases also highlight the potential benefit of the adjusted ARFID protocol in increasing alignment with cultural food practices; this is an important consideration for all of our patients across diagnostic status [19], though remains limited by hospital resources.

This study has several limitations to note, including its retrospective nature and modest sample size which may limit the generalizability of our findings to other inpatient units. Our psychological assessment protocol when patients are hospitalized on our service does not currently provide for the use of standardized measures of ARFID presentations (e.g., with the use of a validated screener), due in part to limitations in staffing and time given the high volume of patients we care for. Although the current study derived clinical assessment information directly from documentation provided by licensed clinical psychologists, future study would benefit from the use of standardized measures. We also do not have longitudinal data; therefore, it is unclear how the adjusted protocol for ARFID impacts medical and psychological outcomes following inpatient discharge, an important focus of future study. While not increasing burden considerably, we also note that the adjusted ARFID protocol requires some additional clinical time and resources for implementation (e.g., caregiver teaching and coordination of menu planning) which may not be feasible in all inpatient settings. Finally, although the case series data presented here suggest that a specialized ARFID protocol will have a positive impact on care trajectory among young people admitted for ARFID, including improved weight gain, reduction of emesis, and improved food intake, prospective studies are needed to compare medical and psychological outcomes to those placed on the standard protocol, and include feasibility and acceptability assessments across patients, caregivers, and clinical providers. Taken together, our descriptive and qualitative findings provide preliminary support for the need for an adjusted protocol for young people receiving inpatient care for ARFID, and for its future empirical evaluation.

## Data Availability

Data used during the current study are available from the corresponding author on reasonable request.

## Abbreviations

ARFID: Avoidant–Restrictive Food Intake Disorder
EBW: Estimated body weight
FBT: Family-based treatment
SAHM: Society for Adolescent Health and Medicine
ASQ: Ask Suicide-Screening Questions
